# Methods for Determination of Antimicrobial Activity of Essential Oils In Vitro—A Review

**DOI:** 10.3390/plants13192784

**Published:** 2024-10-04

**Authors:** Radka Hulankova

**Affiliations:** Department of Hygiene and Technology of Food of Animal Origin and Gastronomy, Faculty of Veterinary Hygiene and Ecology, University of Veterinary Sciences Brno, 612 42 Brno, Czech Republic; hulankovar@vfu.cz

**Keywords:** agar diffusion, agar dilution, antibacterial, biofilm, broth dilution, plant extracts, vapor phase

## Abstract

Essential oils (EOs) have been gaining popularity in the past decades among researchers due to their potential to replace conventional chemicals used in the fight against pests, pathogenic and spoilage microbes, and oxidation processes. EOs are complex mixtures with many chemical components, the content of which depends on many factors—not just the plant genus, species, or subspecies, but also chemotype, locality, climatic conditions, phase of vegetation, method of extraction, and others. Due to this fact, there is still much to study, with antimicrobial effect being one of the key properties of EOs. There are many methods that have been frequently used by researchers for in vitro evaluation; however, although the research has been going on for decades, an internationally accepted standard is still missing. Most of methods are based on time-proven standards used for the testing of antibiotics. Due to the specific properties of EOs and their components, such as volatility and hydrophobicity, many modifications of these standard procedures have been adopted. The aim of this review is to describe the most common methods and their modifications for the testing of antimicrobial properties of EOs and to point out the most controversial variables which can potentially affect results of the assays.

## 1. Introduction

Essential oils (EOs) are mixtures of volatile substances present in various parts of plants, including blossoms (e.g., lavender, orange blossoms, rose, or ylang-ylang), buds (e.g., clove buds), pericarp (e.g., bergamot or grapefruit), whole fruit with seeds (e.g., juniper berries, anise seed, caraway, or cardamom), bark (e.g., cinnamon), wood (e.g., camphor, cedarwood, or sandalwood), rhizomes and roots (e.g., ginger, turmeric or licorice), and predominantly leaves and stems (e.g., eucalyptus, lemongrass, mint, myrtle, oregano, rosemary, sage, tea tree, or thyme) [1,2,3]. In general, the content of EOs in the plant material is very low (less than 5% of the dry matter) [4]. Thanks to their healing properties and pleasant aroma, essential oil-bearing plants have been used throughout the history of humanity to flavor food, cure illnesses, and for spiritual purposes in religious ceremonies [5]. EOs and their main components have been thoroughly studied in the past decades, and their analgesic, sedative, anti-inflammatory, antibacterial, antifungal, antiviral, antioxidant, antiparasitic, and insecticidal properties have been studied and reviewed in detail [2,3,6].

From the chemical point of view, EOs are formed mainly by terpenes and terpenoids, formed in the cytoplasm of plant cells [3]. More than 70,000 different terpenes and terpenoids have been described [4,5]. These substances are also called isoprenoids, as their structure is derived from the isoprene molecule (C_5_H_8_) [4,6]. Whereas basic terpenes are aliphatic or aromatic cyclic (e.g., α-pinene, β-caryophyllene, γ-terpinene, *p*-cymene, limonene) or acyclic (e.g., myrcene) hydrocarbons, terpenoids are modified terpenes with various methyl groups and functional groups containing oxygen. Based on the functional groups, they can be divided into alcohols (e.g., geraniol, linalool, menthol), aldehydes (e.g., citral), epoxides (e.g., β-caryophyllene epoxide, β-cedrene epoxide), esters (e.g., geranyl acetate, linalyl acetate), ether (e.g., 1,8-cineole, anethole), ketones (e.g., carvone), and phenols (e.g., carvarol, thymol). Although there are differences in sensitivity between various bacterial species, the antimicrobial activity generally decreases in order: phenols > aldehydes > ketones > alcohols > esters > hydrocarbons [3,4,5,7]. Terpenes (and subsequently terpenoids) can also be divided into several classes depending on the number of condensed isoprene molecules. Monoterpenes (2 isoprene units) are the predominant components of EOs (90%), followed by sesquiterpenes (3 isoprene units) [3,4,5,6,7]. Monoterpenes and sesquiterpenes are biosynthesised by mevalonic acid pathway in plant cytosol and methylerythritol phosphate pathway in plastids, respectively [8].

The last important class of EOs’ components is phenylpropenes and phenylpropanoids. The name refers to the fact that plants synthesize them from phenylalanine, an aromatic amino acid, by shikimate pathway [8]. Although they are generally less common than terpenes, they may represent the major component in some oils like cinnamon EO or clove EO. The major phenylpropanoids are cinnamaldehyde and eugenol, but cinnamyl alcohol, chavicol, estragole, isoeugenol, safrone, or vanillin also belong into this class [3,5].

EO is generally a mixture of approximately 20–60 chemical substances at various concentrations, but some EOs may contain even several hundred different components [4]. The primary constituents of EOs can make up as much as 85% of the total composition, and, typically, they determine the biological characteristics of EOs. Nevertheless, the remaining 15% consists of minor components. Despite their lower presence, these minor components play a crucial role in biological activities by synergistically interacting with the major constituents [2,4,9].

The practical utilization of EOs is wide—traditionally in perfumery, aromatherapy and pharmacy, currently in both human [10] and veterinary medicine [11]. Their flavoring, antimicrobial, and antioxidant properties also find use in the cosmetics [12] and food and beverage industries [13,14,15]; in agriculture, EOs are used against plant diseases, pests, and weeds [16]. The potential for large-scale application of EOs has been increasing not only due to the higher degree of knowledge and technological advancement, but also thanks to the inclination of part of the population to natural products as alternatives to industrial, synthetized antibiotics (the efficacy of which is threatened by increasing resistance among the target organisms) and food additives (with possible negative side-effects on human health) [17].

**Figure 1:**
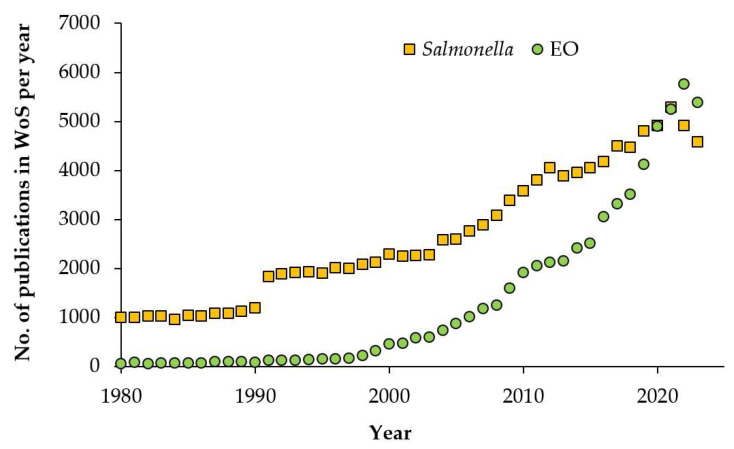
Trend in publications on EOs between 1980 and 2023 with “*Salmonella*” as reference topic (Web of Science survey from 7 November 2024).

Given the number of plant species, subspecies, and chemotypes, there are plentiful possible sources of essential oils with beneficial properties. It has been estimated that about 3000 various EOs are known so far [9], but only a few of them have been scientifically studied, and only about 100 of the most common EOs have their own ISO norm with specified characteristics. The interest in EOs is demonstrated by increasing number of publications per year dedicated to EOs (Figure 1), and establishment of specific scientific journals—Journal of Essential Oil Research (since 1989) and Journal of Essential Oil Bearing Plants (since 1998).

For assessment of efficacy of conventional antibiotics, standardized procedures have been used for years. The antibacterial properties of EOs have been studied for a shorter period of time, and no standardized method has been officially established so far. The researchers usually adapt methods used for antibiotics as described by CLSI (Clinical and Laboratory Standards Institute) or EUCAST for EU (European Committee on Antimicrobial Susceptibility Testing) organizations, intended for determination of susceptibility of microorganisms as surveillance of occurrence and spreading of resistance to antibiotics [18,19,20]. However, lipophilic, volatile substances in EOs may not be suitable for testing by the same methods as conventional antibiotics [19]. Differences in experimental conditions may lead to big variances between results even when using the same method in principle [21]. This can lead to variations in results between research groups [19].

The aim of this review is to summarize the most frequently used methods and their various modifications, analyze the variables and the published data, and suggest the most fitting procedures for analyzing antimicrobial activity of EOs.

## 2. Determination of Minimum Inhibitory Concentration (MIC)

The MIC can be described as the lowest concentration of the tested EO that inhibits the growth of the tested microorganism. MIC determination allows quantitative measurement of the in vitro antimicrobial activity, providing results in easily interpreted units. Knowledge of the effective concentration can be further used in practical application of EO’s in foodstuffs, medicinal products, etc., although it is a well-known fact that due to the complexity of real matrices, the EOs are generally effective in much higher concentrations than in in vitro studies [6,17,22].

The determination is based on incorporation of different (usually two-fold) concentrations of the EO into the medium together with a standardized inoculum of the tested microorganism. The series of EO’s dilutions can be prepared in either a liquid (broth dilution) or solid (agar dilution) non-selective medium. A growth control (without the tested EO) should be always included. Multiple studies also used one or more antibiotics as positive controls [23,24,25,26,27,28,29,30,31]. In broth dilution, negative controls without the testing microorganisms may be used to exclude any contamination during pipetting [32,33,34].

### 2.1. Broth Dilution Method

Broth dilution, and especially microdilution, is a popular method which is highly standardized for testing of antibiotics [18,19,20]. The most important variations of this method include use of solvent (emulsifier), broth type, units, and determination of the MIC endpoint [19,20]. For the macrodilution (tube dilution) method, a volume of 1–10 mL is used, whereas for the microdilution method, a volume ≤ 0.5 mL (most usually 0.1 mL in a 96-well microtitration plate) is used [35,36]. The major drawback of the macrodilution method is the larger amount of EO and medium used, larger space used in the incubators, and greater labor during pipetting due to the impossibility to use a multi-channel pipette, which may easily lead to errors [20]. The methodologies generally originate from standardized methods on testing of antibiotics, where 1 mL is required as the minimum for broth macrodilution [37]. In practice, final volumes of 1 mL [38], 2 mL [39,40,41], or as much as 10 mL [42,43] have been used for the testing of EOs.

**Figure 2:**
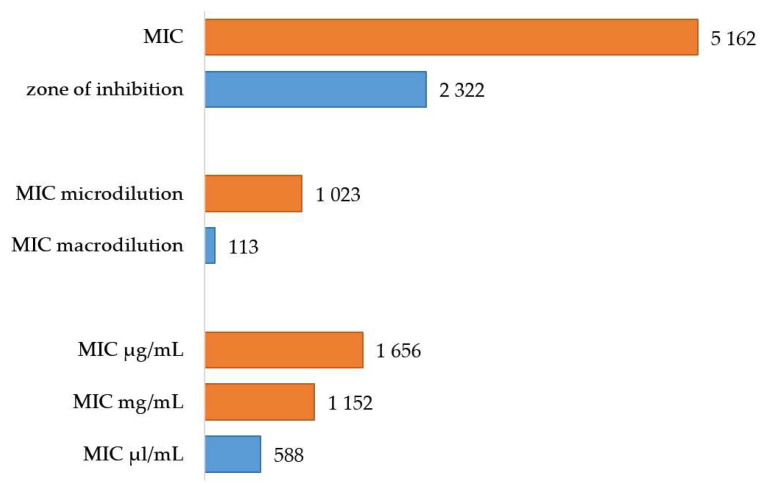
Trends in reporting the antimicrobial effect of EOs (Scopus survey from 7 November 2024). Note that for *v*/*v* units, percentage is frequently used, which cannot be the subject of a search.

Broth microdilution has been much more in use during the past decades (Figure 2), both for economic reasons and reproducibility [20]. However, some studies suggest that the transition of the vapors between wells can affect the microplate assay results [33,40].

Microplates treated with surface coating intended for tissue cultures should be avoided, as such a surface was proven to partially bind the lipophilic antimicrobials, leading to an increased MIC [36,44]. Similarly, microplates with special non-binding, hydrophilic coating showed lower MIC values for lipophilic antibiotics than the other polystyrene microplate types [44].

#### 2.1.1. Inoculum Density

The protocols originate in standardized antibiotic testing [37,45], which has been described in detail in several scientific publications [20,36]. A broth microdilution method specifically for EOs was proposed in 2021 [46] but has not been made into an international standard. Even through the inoculum size may influence the results [21], the vast majority of studies use the final inoculum size 5 × 10^5^ cells/mL, as described in the standards, prepared from a suspension matching the 0.5 McFarland turbidity standard [37,45]. Although in general, the cell density in an assay and the EO’s efficiency are inversely proportional, it may not work the same for all the EOs or their components and for all the bacterial species [21,47].

#### 2.1.2. Type of Broth

Most studies use Mueller–Hinton Broth (MHB) as the cultivating medium for testing the antibacterial properties of EOs [27,28,29,31,44,48,49,50,51,52,53,54,55,56,57,58,59]. The reason is again the CLSI and EUCAST manuals, where cation-adjusted MHB is the mandatory medium for testing resistance of non-fastidious aerobic bacteria to antibiotics [37,60]. However, other authors used also Tryptic Soy Broth (TSB) [32,34,40,46,61,62], Brain Heart Infusion (BHI) [25,33,63,64,65,66], Luria–Bertani broth [30,38,67,68,69,70,71,72,73,74], or Nutrient broth [42,75,76,77,78]. Although TSB showed significantly lower MIC values than MHB (*p* < 0.001) and BHI (*p* = 0.006) in my previous study [79], the differences would be insignificant in a two-fold dilution assay. However, the disadvantage of MHB is that the medium is less nutritious and requires supplementation to allow growth of even mildly fastidious microorganisms, which may pose a problem when comparison of various bacterial species is the aim of study.

#### 2.1.3. Stabilization of the Oil-In-Water Mixture

The use of solvents and/or emulsifiers for dissolving of EOs has been long discussed [9,21,58]. The lipophilic nature of EOs is important for their antimicrobial properties, as it allows them to dissolve in the cell membrane, leading to increased permeability and loss of intracellular matrix, and to pass through the phospholipid bilayer of the bacterial cell membrane, exerting the inhibitory effect on in-cell targets [4,6,17]. On the other hand, the same key feature may lead to problems with dispersion of the hydrophobic compounds in an aqueous phase represented by the cultivation medium. Although some authors did not find the use of dispersing agent necessary [34,51,63,76], especially with constant shaking during incubation [70], most studies used various methods how to ensure the maximum contact of EO with the target bacteria.

Chen et al. [58] studied the dispersion of carvacrol in water and MHB after 2 min and 12 h without shaking. Although carvacrol (unlike the bacterial cells) tended to accumulate in the upper layer, it was visibly much better dispersed in broth than in water, as vigorous shaking in broth led to an emulsion-like solution and only slow separation into a hydrophilic and a hydrophobic phase, especially at lower concentrations. Addition of lysed horse blood (2%), a necessary growth enrichment for fastidious bacteria, was also reported to act as a surfactant [44]. In determining if any stabilizing agent is necessary, the solubility of the major components of the EO in question should be taken into account, as many of the compounds are partially miscible with water (Table 1). Eucalyptol (eucalyptus, rosemary), eugenol (clove), linalool (coriander seed, lavender), carvone (spearmint), and trans-cinnamaldehyde (cinnamon) may not need aid in broth dissolution; carvacrol (oregano) and thymol (thyme) are less soluble; and menthol (peppermint), citral, and limonene (lemongrass, citrus) will need a solvent/stabilizer to prevent stratification in the broth. It is worthwhile to highlight that terpenes are generally less water soluble than terpenoids, and that the solubility may differ between various batches of EO of the same botanical species, as poorly soluble terpenes p-cymene and γ-terpinene are precursors of much-better-soluble phenolic monoterpenoids carvacrol and thymol, respectively, and their ratio may vary between geographical regions, chemotypes, and harvesting seasons [9].

The most common solvents for dilution of EOs are dimethyl sulfoxide (DMSO) [24,27,28,29,30,31,33,52,62,63,64,65,66,69,72,73,78] and ethanol [26,38,48,61,77,81], used alone or in combination with an emulsifier, followed by methanol [23,25,82]. The solvent is usually used only to prepare stock solution of EO, which is further diluted by broth in the assay [24,33,48,54,61,65,70,73,78,82], but some authors spiked the broth instead [27,28,30,31,52,64,72]. Whatever diluent is used, it is crucial to verify that the maximal concentration used in the assay will not interfere with the growth of the tested bacteria by including it in the positive control [30,31,44,58,65,73,83]. In the study by Wadhwani et al. [83], ethanol was found to be the less appropriate solvent, as it was the most inhibitory against the tested bacteria. On the other hand, no difference between the solvents was found in agar dilution for *Escherichia coli* [58]. However, both studies suggest to decrease the final concentration of the solvent in any assay as much as possible, with the recommended value ≤ 1%, in order to prevent potentiation of antimicrobial effect of the tested EO. Although many studies are within these parameters [24,27,33,64,65,68,73], some used 2% [84], 3% [52], 4.4% [29], or even 5% of DMSO [30,31,72] as the final concentration. The antimicrobial effect of the solvent may be species-dependent and should be verified in advance, especially for the testing of fastidious species.

Non-ionic surfactants can also stabilize the emulsions of hydrophobic EOs. Polysorbate 80 (Tween 80) [32,40,44,46,53,54,69,85,86,87,88] has been used in many studies as emulsifier, either alone or in combination with solvents [69]. Deceleration of the separation process of EO from the water phase enables a more efficient inactivation of bacterial cells [47]. It was also suggested that Tween 80 reduced binding of the lipophilic antibiotics to the plastic surface of the microtiter plate [44]. However, 0.5% of Tween 80 had inhibitory effect on *Helicobacter pylori* [86]. Nielsen et al. [86] noted a stimulating effect of Tween 80 itself on growth of *Staphylococcus aureus* and inhibitory effect to *Pseudomonas fluorescens* in a concentration of 0.1%. It was also stipulated that Tween 80, at higher concentrations, forms such small micelles that EOs get entrapped in and which are not in contact with bacteria, whereas the bigger droplets are more effective [21,47]. On the contrary, Tween 80 and Tween 20 has been repeatedly used to create micro- and nanoemulsions of EOs which showed higher antimicrobial efficacy than the EO itself, the efficacy increasing with decreasing size of droplets, and higher concentration of the emulsifier generally creating smaller droplets [88,89,90]. In addition to this controversy in using Tween, the emulsion may show an increased turbidity, which can pose a problem for assays measuring optical density [9].

In order to avoid the possible interactions of EOs or bacteria with solvents and detergents, addition of agar in a concentration of 0.15% was used in multiple studies to stabilize the dispersion [9,42,50,59,67,68,69]. The methodology was introduced by Remmal et al., who produced a stable, homogenous dispersion using a broth with 0.2% of agar [40,91]. The MIC in assay with agar was significantly lower in comparison to Tween 80 (0.25%) or ethanol (0.2%). Similarly, Mann and Markham [92] reported no evident separation of tea tree EO from a nutrient broth supplemented with 0.15% of agar, in comparison to 0.2% of DMSO, 2% of ethanol, or 0.5% of Tween. In the study by Thielmann et al. [29], agarose (0.15%) was used instead of agar. Unfortunately, suspensions of some EOs were clouded, and DMSO had to be used instead. Similarly, Bouyahaya et al. [68] used agar to stabilize EO for MIC determination by broth microdilution, but for the kinetic measures based on optical density, the EO was diluted in DMSO instead.

Emulsification may be also achieved by sonication [46,56,93]. The method is often part of preparation of EOs’ nanoemulsions. Ultrasound waves create local pressure and turbulences in the liquid, leading to collapse of larger oil droplets and creation of multiple droplets with smaller diameter [90,93].

#### 2.1.4. Endpoint Determination

Large variability is in how to determine the endpoint—the concentration at which the tested microorganism does not grow. Many authors use visual determination of the MIC (bacterial growth), as determined by pellet formation or visible turbidity of the liquid medium [32,34,42,52,53,75,77,79,82,88]. For a pellet formation, microtiter plates with U-shaped bottom should be used, without rotation/shaking during incubation. Unfortunately, some fastidious, microaerophilic bacteria like *Helicobacter* spp. may not grow abundantly in a liquid medium, thus making growth detection by the naked eye unfeasible [93], or they require an addition of lysed blood for their growth, which darkens the medium and makes the reading difficult. Even for non-fastidious bacteria, it may be hard sometimes to say if the medium is really clear or very slightly hazed.

**Figure 3:**
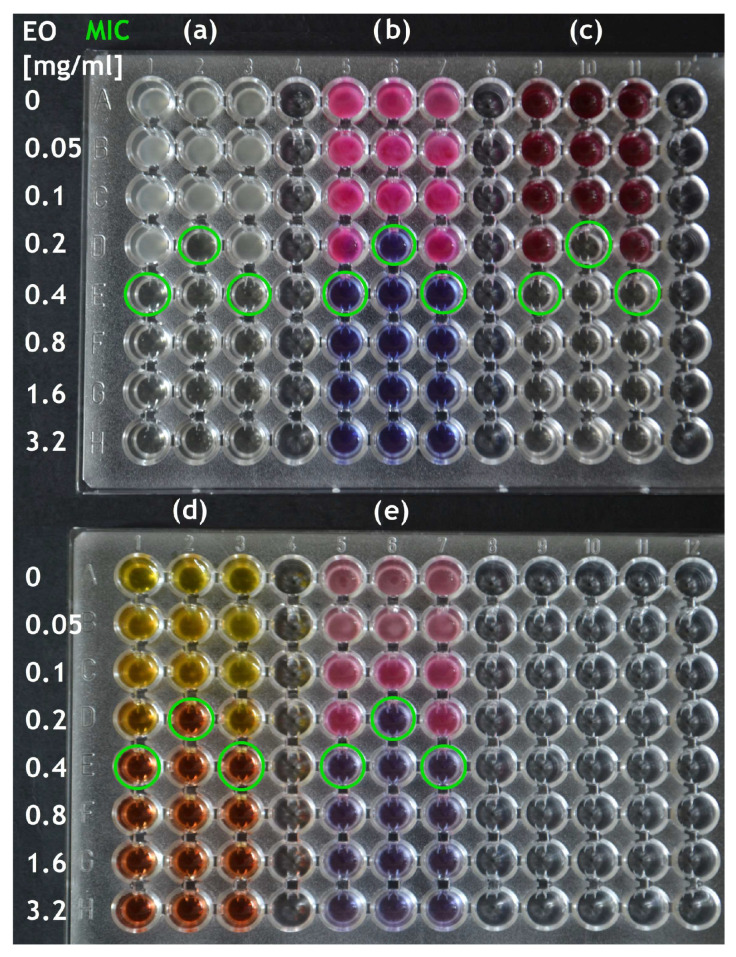
Examples of broth microdilution assays used for testing of EOs with different end point determination: (**a**) MHB with 0.15% of agar (mMHB); (**b**) mMHB with resazurin added after incubation; (**c**) mMHB with tetrazolium (INT); (**d**) mMHB with phenol red; (**e**) mMHB with resazurin added before incubation.

For easier and more precise MIC determination, various colorimetric assays with a redox indicator have been published (Figure 3). Resazurin is used most often [24,46,50,55,58,59,68,78,92], based on the principle of its reduction to fluorescent resorufin by mitochondrial enzymes of metabolically active cells [50,94]. The addition varies from 5 to 30 μL per 100 μL with a final concentration of 0.001–0.002% [46,50,55,58,59,68,92]. The dye is added after incubation, and the assay is further incubated usually for 2 h [50,59,68,92], although 0.5 h [46], 1 h [58], or even 2–4 h [55] were reported in various studies. Alternatively, 10 μL of resazurin solution (270 mg in 40 mL) can be added prior incubation [24,78,94]. This procedure is more practical, since the dye can be pre-mixed into a medium used for preparation of EO solutions, reducing the necessity to mix each well. Color changes from blue (negative) to pink (positive), while discoloration indicates a reversible reduction of resorufin to dihydroresorufin [92]. The color change is easy to read by the naked eye or by a microplate reader measuring absorbance or fluorescence [92,95]. However, fluorescence reading is possible only in assays where resazurin is added post incubation, since the colorless dihydroresorufin formed after prolonged incubation does not fluoresce [94].

Tetrazolium assays are about as much in use as the ones with resazurin. The principle is a transformation of a more-or-less colorless tetrazolium salt to a brightly colored, water-insoluble formazan by reducing cellular (predominantly mitochondrial again) enzymes (oxidoreductases and dehydrogenases), thus indicating respiratory activity [48,95,96]. Tetrazolium chloride (tetrazolium red, TTC) has historically been most used [26,38,54,81,96], followed by tetrazolium bromide (thiazolyl blue, MTT) [33,96,97], and iodonitrotetrazolium chloride (iodonitrotetrazolium violet, INT) [28,30,31,48,93,96,98]. The color change in tetrazolium usually takes place within 10–60 min [96]. Although Klančnik et al. [48] did not find any differences between TTC and INT, they recommend INT to indicate the viability of aerobic bacteria. In the study by Ellof [96], INT was also found to be the best indicator, as its formazan stayed stable, and there was no color change in negative control. Moreover, INT and MTT worked at a concentration of 0.2 mg/mL, which was ten times lower than the minimal working concentration for TTC [48,96]. As is the case of solvents and detergents, toxicity of the chosen dye should be checked in advance for testing of nonstandard, fastidious bacteria, for which tetrazolium or resazurin may be toxic [94]. Negative control is also very important, as the antioxidant (reducing) potential of EOs could in theory affect the results [48,95].

Several authors also used Mueller–Hinton broth supplemented with 0.02 g/L of phenol red [85,87,99], which works as a pH indicator, changing color during incubation from red (negative; alkaline pH) to yellow (positive; acidic pH). The color change would be enhanced due to absence of buffering compounds in Mueller–Hinton broth. Knezevic et al. [64] added Christensen’s urea broth into BHI after incubation and incubated the assay for the next 4 h for MIC determination of various EOs against *Helicobacter pylori*.

Klančnik et al. [48] used ATP activity to determine MIC of plant extracts against *Campylobacter* spp., obtaining results comparable to the INT and TTC assays. Inhibition of intracellular ATP synthesis and leakage of ATP outside the bacterial cell is one of the mechanisms of the antimicrobial effect of EOs [3,4,7]. A commercial reagent causing cell lysis and oxidation of luciferin by luciferase, followed by measurement of luminescence by a microplate reader was used in the study [48].

The last-but-not-least-common method is spectrophotometric measurement of optical density (OD). A minor variability exists in published wavelength, using 600 nm [40,62,64,65,71,76], but also, for example, 531 nm [100], 570 nm [101], 620 nm [23], or 655 nm [67], depending on the instrument. Donaldson et al. [40] found MICs from INT and OD assays highly correlated, when determining activity of multiple EOs. MIC is defined as the lowest concentration at which OD does not increase after incubation in comparison to initial measurement [47], although the percentage of inhibition (50, 90, or 100%) in comparison to the control reading has been computed instead in several studies [40,100,101].

#### 2.1.5. Units of MIC

Based on the survey of Scopus database, MIC is mostly expressed in mg or μg/mL (*w*/*v*), although many studies use μL/mL or percentage (*v*/*v*) (Figure 2). Using different units makes it difficult to compare MICs from different studies. While using *v*/*v* units based on pipetted volumes is easier for the researchers, *w*/*v* represent a more precise result. The conversion of units is based on density of EO, which is unfortunately not listed in each study. In one of the key reviews on EOs [9], the results were all converted to μL/mL for easier comparison, assuming the same density of EOs as water. The relative density of EOs at 15 °C varies between 0.696 and 1.118 [102]. While most of the EOs are less dense than water, cinnamon EO, clove EO, and sassafras EO show a higher density [103]. Although the results will not differ by an order of magnitude, the unification of units would help in cross-study comparisons.

### 2.2. Agar Dilution Method

The principle of the method is mixing diluted EO at different concentrations with defined amount of melted agar; after agar solidification, a specific number of bacterial cells are spot-inoculated onto the agar surface [6,20,36]. As with the broth dilution, the MIC is the lowest concentration that totally inhibits the bacterial growth [37].

Similar to broth dilution, the most important variations in this method include use of solvent/emulsifier, agar type, and units. Inoculum is usually used according to official guidelines for antibiotic testing. Briefly, a bacterial suspension of approx. 10^8^ cells/mL is prepared using 0.5 McFarland turbidity standard/OD measurement, tenfold-diluted, and 1–2 μL are spot-inoculated on agar, leading to approx. 10^4^ cells being applied per a spot. The inoculum is not to be diluted if a replicator with smaller pins (dispensing only 0.1–0.2 μL) is used [37]. The same standard was kept in miniaturized methods [58,104], but in some of the 24-well plate methodologies, each well was inoculated with 20 and 50 μL, respectively [105,106].

Although most of studies use a Mueller–Hinton agar (MHA) as the cultivation medium [58,104,107,108,109,110], some researchers have used Tryptic Soy agar (TSA) [106,111,112] or BHI agar [113,114], either alone or nutrients-supplemented in the case of fastidious bacteria. Similarly to the broth dilution, some studies use solvents/emulsifiers for even distribution of EO in the agar, namely 1–2% of DMSO [104,113] or 0.5% of Tween [109,110]. The question is how much their presence is needed in a solid medium, with the agar itself working as a stabilizer, as described in the chapter about broth dilution. However, the solvents may still be useful for preparation of the stock solution of EOs.

**Figure 4:**
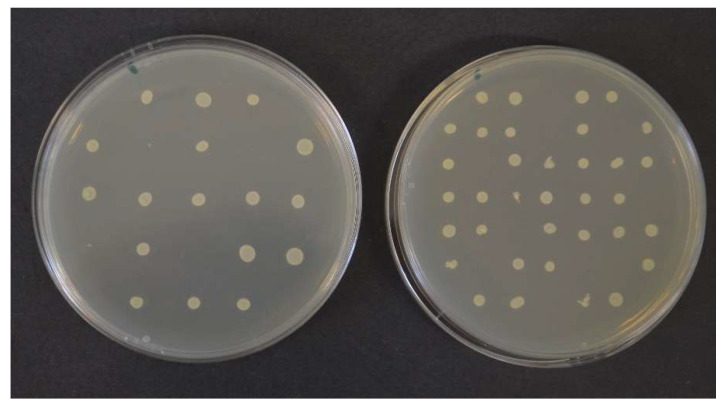
Examples of agar dilution assays with visible growth/inhibition of the tested strains (1 μL of inoculum).

The method has several advantages and disadvantages to consider before use. Given the method of inoculation, it allows for testing of multiple bacterial strains using only one set of plates. When using a multi-point inoculator, up to 60 strains can be inoculated on a 90 mm plate [20], and about half the amount by hand-pipetting technique (Figure 4). This ensures identical testing conditions suitable for strain comparison when testing a single EO or EO component [36]; however, for testing of multiple EOs on a small amount of bacterial strains, disk diffusion or broth dilution may be a better choice. The second major advantage is oxygen or other gasses (CO_2_, N_2_, H_2_) availability, making agar dilution more suitable for fastidious anaerobic or microaerophilic bacteria (e.g., *Helicobacter pylori*, *Campylobacter* spp., *Neisseria gonorrhoeae*), which may grow more poorly in broth dilution (especially macrodilution), where the bacteria are dependent on the dissolution of gasses into the liquid medium [6,20,21]. What more, necessary incorporation of growth promoters (blood, vitamins) into agar has no effect on MIC readability, as well as natural opacity or coloring of natural substances including EOs [6,20,102].

Among disadvantages, the larger used-up volume of EO and medium is most frequently mentioned. When using Petri dishes 100 mm in diameter, sufficient amount of agar must be added (about 3–4 mm in height, corresponding to 25 mL) to create a sufficient layer resistant to desiccation [36]. For a 90 mm Petri dish, 20 mL is standardly used [58]. This means that a higher amount of EO is used up to prepare a set of plates with different concentrations in comparison to broth dilution, not to mention broth microdilution [36]. The amount of the material may be cut down by miniaturization. An agar mini-dilution method for determination of MIC of EOs has been recently published [58]. The method used 35 mm Petri dishes with the final volume of 6 mL, mixing 3 mL of double strength MHA with 3 mL of diluted EO. Agar microdilution method of Golus et al. [104] used mixing of EO and MHA in Eppendorf tubes before dispensing 100 μL into a 96-well microplate, with inoculation of one strain per well using a multichannel pipette. Some authors adopted an agar dilution method using 24-well plates [105,106,107,108,111,112,113] with the final volume of 500 μL and inoculated with a single strain.

Unlike in broth dilution, the agar must be tempered to 45–50 °C [36,37] to stay liquid, which means that the temperature of EO after mixing is increased. In the agar microdilution method [104], the mixture was kept at 50 °C in a ThermoMixer before dispensing. Temperature can influence some physicochemical properties of EOs, including vapor pressure. Since EOs with different compositions may differ in volatility, increased temperature especially with agitation could enhance vaporization and loss of the highly volatile EO components [21]. The recommended agar temperature should not be exceeded, and for comparison of different Eos, other methods of MIC determination should be considered, although, of course, some evaporation during incubation will occur even in broth dilution [33].

The agar microdilution still has a great potential as a rapid and cheap method, as it was reported to show higher accuracy and reliability than the broth microdilution or agar dilution method [58] and generally slightly lower MIC values in comparison to broth microdilution. The authors suggested that due to the small amount of agar in the well (100 μL), the medium solidified sooner than any separation of EO and water phase could occur [104].

## 3. Determination of Minimum Bactericidal Concentration (MBC)

Determination of MBC is usually used as a complementary measurement to the determination of antimicrobial activity by MIC through broth dilution. It can be defined as the minimum concentration of EO which devitalizes the tested microorganism. The usual method of determination is based on plating out 2–100 μL (most frequently 10 μL) from each tube/well, showing negative visible growth after incubation, as the MBC can be either equal to or higher than MIC. The agar for plating usually corresponds to the broth used for MIC determination [23,24,25,26,28,32,50,51,52,53,61,62,65,67,68,71,72,75,81,82,85,93,98]. Sub-cultivation of 100-fold dilutions in broth instead of plating on agar has been described, too [77]. By checking for visible growth in medium without the tested EO, it can be determined if the microorganism was lethally damaged or only inhibited but still able to multiply under more favorable conditions. The most precise method is based on enumeration of initial inoculum by plating of serial dilutions, and its comparison to CFU counts in various EO’s concentrations from MIC determination. The MBC is determined as the lowest concentration decreasing the initial inoculum by 99% [70], although the definition by CLSI gives the value 99.9% [20,47].

## 4. Kinetic Studies

Kinetic studies (time-kill assays, survival curves, curve-assays) are used to determine the time needed for total elimination of the tested microorganism and to validate the MIC and MBC values. Kinetic studies may include growth curves, using concentrations lower than MIC (e.g., 1/2, 1/4) [19,62,68,71,106], or inactivation curves, using the concentrations around MIC or higher (e.g., MIC, 2× MIC) [42,48,59,62,71,99,107,109].

The easiest way to conduct growth kinetic studies is to use a microplate reader, reading OD in the liquid medium during incubation at pre-set time intervals [19,62,65,68,71,74,106]. Alternatively, a small amount of the medium is diluted, plated out, and enumerated at several times [20,24,42,48,59,71,100,107]. While this method is much more laborious in comparison to OD measurement, it is actually the only way how to obtain the inactivation curves.

## 5. Agar Diffusion Methods

Diffusion methods are usually used as preliminary screening methods to determine if the tested EOs have at least some antimicrobial activity. By testing multiple EOs in a rapid, easy, and cheap manner, EOs with very-low-to-no antimicrobial activity can be eliminated from further testing. Since the result is rather semi-quantitative, it is strongly recommended to follow up with MIC determination. The diffusion method is considered less comparable between various studies on EOs than MIC, for parameters such as agar thickness and amount of EO vary considerably [9,21].

**Figure 5:**
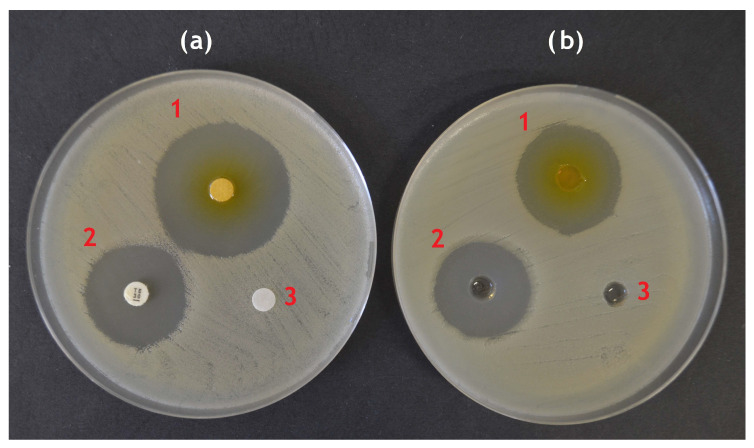
Examples of agar diffusion assays with zones of inhibition: (**a**) agar disk diffusion; (**b**) agar well diffusion; 1 = cinnamon EO 10 μL (**a**) and 20 μL (**b**), 2 = positive control (tetracycline disk/solution), 3 = negative control (sterile distilled water). Disc/well diameter: 6 mm.

The principle of the assay is diffusion of the antimicrobial components of EO into agar plates inoculated with test strain, inhibiting the growth of the test strain around the spot (disk or well) where the EO was applied. Both positive (a conventional antibiotic disk/solution) and negative (sterile distilled water) control should be used. The plates are left alone for 30 min to allow the EO to diffuse before incubation. After incubation, a zone of inhibition is created (Figure 5), and its diameter is measured and reported in mm [20,22,47,96]. However, the size of inhibition zone has no practical use, as it does not indicate which concentration can be effective in applied research (food model, medical treatment, etc.). Although the MIC can be calculated from the zone of inhibition size (and vice versa) for common antibiotics [20], no such algorithm has been validated for EOs. Several authors used the agar diffusion method to determine MIC, using not the pure EO, but its dilutions. The MIC was defined as the lowest concentration of EO which produced an inhibition zone after incubation [48,88,115]. However, the MIC obtained by agar diffusion were reported to be generally higher that the MIC obtained by dilution methods [48]. In many studies, the zone of inhibition size did not even correlate with MIC values [40,50,66,77,96]. The commonly used explanation is that the EOs diffuse differently into the agar depending on their polarity. The EOs richer in compounds with better water solubility (Table 1) will diffuse more quickly and create larger inhibition zones, whereas the hydrophobic compounds would tend to remain on the agar surface and gradually evaporate during incubation. Thus, even if the zone of inhibition is not created or is small, the EO may be in fact still effective [47,48]. Due to the volatile nature of EOs, many authors do not consider agar diffusion methods suitable for testing of EOs at all [48,96,107].

It should be noted that for screening purposes, some authors use the sensitivity of EO evaluation based on the size of the inhibition zone. It would be better described as the EO’s efficiency or strain sensitivity. The EOs with inhibition zone diameter ≤ 8 mm can be considered non-efficient, whereas the EOs with an inhibition zone ≥ 15 mm are considered very efficient, and ≥20 mm is considered extremely efficient [30,55,57,116]. On the other hand, another highly cited study used the limits for zone of inhibition < 12 mm (not inhibitory EO) and ≥ 20 mm for strongly inhibitory EO [117]. Although slightly different in the methodology, both original publications use 6 mm disks [116,117].

Last but not least, the zone of inhibition was measured from the edge of the disk/hole to the outer edge of the inhibition zone in one study [40], whereas in the others, it was measured as a diameter or was not specified. The diameter is the standard measurement for the Kirby–Bauer method according to CLSI and EUCAST [60,118], whereas the other definition of inhibition zone is used in detection of residues of antibiotics in animal food products by plate method. Since the diameter of the zone of inhibition is obviously primarily affected by the disk/hole diameter, any variability in disk/hole size makes for difficult comparability between studies.

### 5.1. Disc Diffusion Method

Generally, there are two types of diffusion methods: disk diffusion and well diffusion. The disc diffusion method is much in use, since it is rapid, easy to perform, and standardized for antibiotic testing. Both CLSI [118] and EUCAST [60] use it as the reference method for both common and certain fastidious microorganisms. Although the method originated in the 1940s [20], it has been validated 20 years later and became known as Kirby–Bauer method [118]. Both CLSI and EUCAST prescribe the use of MHA, if needed, supplemented with either 5% of sheep blood [118] or horse blood and β-NAD [60]. Most studies on EOs also use MHA [23,40,48,49,50,51,53,54,67,107,109,117]; other media such as nutrient agar [88], Luria–Bertani agar [30,31,98], or BHI agar [27,119] are rarely used. Fastidious anaerobe agar is recommended for testing of anaerobes by the EUCAST manual [60]. The manuals offer some level of standardization for the agar depth (approx. 4 mm) and inoculum size (McFarland standard 0.5, corresponding to approx. 10^8^ cells/mL). However, the disk impregnation with the EO is left to the imagination.

When testing EO, pure oil is applied on a paper disk, although some authors used dilutions of specific weight of EO in ethanol [23], methanol [25], DMSO [28], or water with DSMO with Tween 80 for better diffusion [112]. A 6 mm paper disk can be considered the standard [23,28,30,31,48,49,50,53,54,63,67,78,88,98,109,119,120], but 5 mm [107], 7 mm [115,121], 8 mm [40], or even 10 mm [51] have been occasionally used. One study mentioned using three layers of the filter paper [49], while the other studies seem to use only one layer. Most of studies only mention filter paper, but a few studies specify it as grade 1 [75,78], and others specify it as grade 3 [121] or 5 [109]. Most importantly, even the volume of EO applied onto the disk varies a lot among the studies, with no connection with disk diameter. A 6 mm disk with 10 μL of EO seems to be the standard [30,31,48,50,53,54,63,98,119,120], although 4 μL [107], 5 μL [67,121], 15 μL [23,40], or 20 μL [25,49,78,88,115] were used in other studies. Dipping of the disk into EO should be totally avoided, as the volume of EO soaked up by the paper cannot be regulated.

Given the above-mentioned variables, the sizes of inhibition zones are entirely incomparable between different studies. Table 2 shows the suggested parameters for both dilution and diffusion methods in order to improve their standardization.

### 5.2. Well-Diffusion Method

This variant of agar diffusion is less in use, since it is more laborious than the disk method. Instead of placing paper disks on the agar surface, wells/holes are drilled out into the agar and filled with EO or its solution. A sterile borer can be used or replaced by a sterile pipette tip with a sharp rim. The agar plugs need to be efficiently removed out of the wells. Some authors use Oxford cups to either pour agar around to create the wells [68], or the cylinders are simply placed on top of the agar plate and filled with EO [77]. The inhibition zone is affected by both the diameter and depth of the well. Furthermore, during drilling, the agar layer may separate from the bottom of agar plate, resulting in leakage of EO from the well. The bottom of the well thus should be sealed by a few drops of molten agar before application of EO [59], which makes the whole method even more laborious.

The main variables include hole diameter, volume of EO tested, and medium. Although the most common hole diameter is 6 mm, as for the disk method [38,70,77,88], use of 4 mm [40], 7 mm [57,59,115], and 8 mm [68] wells have been published. Besides MHA [40,57,59,115], many authors used Luria–Bertani agar [38,68,70,97] or nutrient agar [77,88]. The volume of EO include 10 [59,97] or 15 μL [40] but also larger volumes such as 50 μL [57,68,88,115], 80 μL [70], 100 μL [38], and even 200 μL [77]. However, the larger volumes are possible only when using Oxford cups.

Although results of disk and well diffusion method strongly correlate [40], the level of diffusion and vaporization can be slightly different, creating different concentration gradient and not identical size of inhibition zone (Figure 5, ref. [40]).

## 6. Antimicrobial Activity in Vapor Phase

Due to the high volatility of EO’s components at ambient temperature, they exhibit bioactivity in the vapor phase [33]. This is advantageous for developing inhalation therapies, for room and air disinfection, sterilization and protection of stored fresh produce (e.g., in controlled atmosphere), and for extending the shelf-life of foodstuffs by active packaging [47,121]. Incorporating EOs into a biopolymer matrix offers several benefits, such as avoiding labelling of EOs as food additives, and reduces the negative impact of the EO on the sensory properties of the product [5,22,121]. However, similar to their liquid-phase counterparts, vapors are less effective in food than in vitro [122].

Several studies have reported morphological changes in bacteria exposed to EOs’ vapors, including reduced membrane potential, decreased intracellular pH, increased cell permeability, and loss of intracellular ATP, mirroring the effects observed in the liquid phase [122].

Although many studies concluded that EOs were more potent inhibitors in vapor form [5,70,122,123], others found it quite the opposite [120,124]. One suggested reason for the increased efficacy in the vapor phase is that lipophilic molecules cumulate to form micelles in the aqueous phase, which limits the contact of the EOs with the microorganism, whereas the vapor phase offers full contact. The antimicrobial activity in the vapor phase is attributed to the presence of highly volatile compounds. It was found that using the disk diffusion method, only water-soluble molecules diffused deeply into the agar, while the other molecules created inhibitory effect by redeposition on the agar surface after previous vaporization. EOs containing alcohols, ketones, esters, oxides, and hydrocarbons showed more distinctive inhibition from vapors, whereas EOs with higher aldehyde content showed inhibition primarily through diffusion [124]. Therefore, assessing the antimicrobial properties in both liquid and vapor phase can provide valuable data.

Despite this, the liquid phase is predominantly used in the studies instead of the vapor phase. Susceptibility testing of microorganisms to EOs and their volatile components using standard broth and agar methods is arduous due to their high volatility, viscosity, and hydrophobicity. Unlike the standardized methods for antimicrobial susceptibility of conventional antibiotics, there are no such assays for volatile compounds in the vapor phase [33,124].

The vapor phase assay for EOs, first described by López et al. [123], is based on disk diffusion method. The agar plate is prepared in the same way, but the EOs are added to sterile filter disks placed on the inner side of the lid of each Petri dish, which are incubated bottom up. Some modifications have been made to avoid direct contact between the essential oils and the plastic lid [47,122]. As with the disk diffusion, undiluted EO is used and the diameter of zone of inhibition measured [120,124]. Alternatively, several plates with dilutions of EO are prepared, and the MIC is determined as the lowest concentration that produces a visible inhibition zone [47]. The MIC (or MID, the Minimum Inhibitory Dose) is expressed in μL of EO per volume unit of air inside the Petri dish [47,122,123,124]. The volume of free atmosphere must be calculated depending on the dish diameter and thickness of agar layer, and the Petri dish is sealed with parafilm to minimize the leakage. It should be noted that for this purpose, sealing is sometimes, but not always, noted also in direct contact agar diffusion studies [38,104,109,120].

A four-sectioned Petri dish adaptation of the disk volatilization method has been proposed, allowing for testing of four microorganisms on the same plate [125]. More recently, broth-microdilution- [126] and -macrodilution [33]–volatilization assays were published, combining broth dilution and disk volatilization methods. Whereas in the wells of microplate, the EO was tested in liquid phase; a small amount of agar was pipetted on the lid for testing in vapor phase. The growth was visualized by MTT dying. However, since there were some drawbacks in the microdilution, macrodilution was proposed [33], using 2 mL Eppendorf tubes. The results were not affected by smaller amounts of agar on the microplate lid in comparison with the tube cap, and the vapors could not affect neighboring wells.

Since the methods of testing in the vapor phase have been thoroughly reviewed recently [121], with 11 different procedures found in the literature described, more detailed information was not included into this review.

## 7. Antimicrobial Activity against Biofilm Formation

Biofilms are specific microbial communities attached to biotic or abiotic surfaces [127]. Bacterial cells in biofilm were found to be extremely resistant to unfavorable environmental conditions, including treatment by antimicrobial compounds [25,78]. Biofilm formation is a major problem not only in medicine and medical facilities but also in the food industry, as many foodborne pathogens are able to persist on surfaces, leading to cross-contamination and increased risk of foodborne outbreaks [88,122,127].

It is no wonder that biofilm removal and prevention of its formation are important aims in studying antimicrobial properties of EOs. The principle of the anti-biofilm effect is mostly being connected with quorum sensing. EOs were found to inhibit expression of several genes responsible for adhesion, decrease metabolic activity of bacterial cells and the production of proteins and polysaccharides forming the extracellular matrix [71,73,88,128,129]. EOs with anti-biofilm activity have been reviewed, e.g., by Nuță et al. [130] or Nourbakhsh et al. [131]. However, it should be noted that any effect may be strain-dependent. Artini et al. [132] performed a large study on various *Pseudomonas aeruginosa* isolates with 61 EOs at the same concentration 1% *v*/*v*. The composition of individual EOs was evaluated with respect to inhibition/stimulation of biofilm formation, using machine-learning algorithms. The percentage of biofilm formation in comparison to control varied to a great extent between strains, and in many cases even stimulating effect (>120%) was detected.

Most of the studies reported both inhibited biofilm formation/cell adhesion in the presence of EOs [25,27,68,78,88,128,129,133] and successful removal of pre-formed biofilm [25,38,49,68,78,88,128,129]. In the study by da Silva et al. [88] on *Listeria monocytogenes*, *S. aureus*, *Salmonella* spp. and *E. coli*, oregano EO, thymol, and carvacrol decreased the adhesion by approx. 49 to 99% (concentration ½ × MIC), while the removal of adherent cells ranged from approx. 65 to 92% (concentration 2 × MIC). Kavanaugh et al. [49] also reported that for some EOs, *Pseudomonas* biofilm was more susceptible than planktonic cells. On the other hand, other studies demonstrated higher resistance of biofilms in comparison to planktonic cells [27]. However, while the MIC has some merit in comparison of biofilm formation and planktonic growth inhibition, it is not very valid for biofilm removal, where the MBC value for planktonic cells (which is not always determined) makes much more sense as the testing concentration.

Since MIC and its multiples are used for biofilm testing, a standardized method of MIC determination has its importance also here. As for methods of biofilm testing, standard methods seem to be used without specific modifications for EO testing. However, as for the previous testing methods, some authors also used solvent or emulsifiers to increase the stability of EOs in the hydrophilic environment [25,49,88]. Nielsen et al. [86] found that Tween 80 itself led to formation of less biofilm by both *L. monocytogenes* and *Pseudomonas fluorescens* and decreased the effectivity of isoeugenol.

The methods for biofilm testing have been revised previously [127,134]. Static methods for forming biofilms are highly favored due to their simplicity, high reproducibility, and cost-efficiency. However, these methods have several drawbacks, including the inability to provide a continuous supply of fresh medium and inadequate aeration [127]. Based on literature survey, microplate assay has been used most for testing of EOs, using plastic plates intended for tissue culture. The bacterial culture is either incubated directly with EO (at MIC multiples) to measure the potential to prevent biofilm formation in comparison to control, or, to measure the potential for biofilm removal, after biofilm formation, the wells are carefully washed out to remove waste products and planktonic cells, and a fresh medium with EO (at MIC multiples) is added in the next step. In both methods, after incubation, the cells in biofilm attached to the pore wall of the well are stained mostly by crystal violet solution, and the coloration is checked visually or mostly by absorbance measuring at 570, 590 or 595 nm. For measurement, after rinsing and fixation the bound crystal violet is dissolved to form a homogenously colored solution fit for measuring [25,27,38,62,68,72,78,88,112,128,129,132,133]. Some studies detected the metabolic (respiratory) activity by dyeing the cells with tetrazolium salts [25,27,65,73,129], using the wavelength 570 nm, or resazurin [128,129]. Based on the control OD values, the percentage of biofilm inhibition/removal is calculated.

Calgary biofilm assay is an alternative to a classical microplate method. In general, the assay consists of a microtiter plate with a lid with special plastic pegs attached, so after placing the lid, each peg is immersed into a microtiter well containing inoculated broth. During incubation with shaking, the biofilm is formed on the peg. The lid is transferred to another microtiter plate for testing of the antimicrobial compound. This way the planktonic cells are easily removed from the assay and the biofilm is not disturbed by washing processes [127,134]. Kavanaugh et al. [49] used a commercial assay for testing of various EOs and EOs’ components and calculated MBEC (minimal biofilm eradication concentration, in %) as an equivalent of the MBC. The cells were released from biofilm by sonication and enumerated by plating. The MIC also has an equivalent in minimum biofilm inhibition concentration (MBIC) [129].

Decreased motility, such as twitching, swimming, and swarming can be detected by cultivation on semi-solid agars [72,73,78,112,128,133,135]. Motility, connected with the presence and correct functioning of pili and flagellae, is not just a virulence factor but a necessity for adherence and biofilm formation. Another parameter connected with adhesion is cell–surface hydrophobicity. The principle is mixing bacterial culture with hydrophobic hydrocarbon, usually xylene. OD in aqueous phase is measured after its separation and compared to OD of the initial suspension. Reduced hydrophobic capacity impairs adhesion to surfaces and biofilm formation [73,78,119,135]. Many studies also measure auto-aggregation ability, based on OD measurement of liquid static culture [73,129,135]. Impaired adhesion and biofilm formation can also be corroborated by down-regulation of expression of biofilm-related genes [73,119,128,129,136]

Testing of biofilm formation/removal on pieces of specific materials have been used far less than testing in polystyrene microplates. The methodologies include both prevention of biofilm formation (simultaneous addition of EO and bacterial culture), or inhibition of previously created biofilm. The principles are the same as for polystyrene microplates, but biofilm is grown on previously sterilized “chips” or “coupons” (pieces such as 2 × 2 cm^2^). The pieces are treated with tested EO at multiples of MBIC, and the biofilm mass determined at several time intervals. Although medical devices such as catheters or implants have been tested, too [130], most studies focus on stainless steel [25,65,66,72,73,128,129,136,137], glass [66,73,128,129], or various plastic surfaces, such as nylon, silicone, polyurethane, HDPE, PET, PP, or PTFE [25,66,73,128,136]. The hydrophobicity of the material plays a role, with most of plastic surfaces being more hydrophobic than steel and, in particular, glass. Generally, biofilm is more easily formed on plastics, but the reduction in comparison to control is also the greatest, although physicochemical characteristics of individual plastics also play a role [66,73,128].

Based on the literature survey, there are variations in the incubation conditions for adequate biofilm formation—e.g., from 6 h [25] to 2 d [88,129] for *L. monocytogenes*. Various media have been used in the assays, such as MHB [27,49,88], Luria–Bertani broth [38,68,71,72,130], TSB [27,78,129], or BHI [119,132]. However, with percentage of inhibition being a relative value counted from control, the results are still more comparable between studies than the MIC/MBC values for planktonic cells.

## 8. Other Methods Used in Testing of EOs’ Antimicrobial Activities

Since the phenolic components of EOs are known as membrane permeabilizers, microscopy plays a significant role in observing the structural damage of membranes and cell lysis of both planktonic cells and biofilm caused by EOs. Scanning-electron microscopy (SEM) [71,106,112,128,129,133], transmission electron microscopy (TEM) [78,112,133], and confocal microscopy (CLSM/LSCM) [62,65,66,71,86,106,128,129,133] have been used in many studies for this purpose. The microscopic techniques have been well described, for example, in a review by Azeredo et al. [134].

Structural changes to membranes can also be detected by measuring membrane potential using a fluorescent probe [62,65,71,106], leakage of nucleic acids and/or proteins [68,73,106,112,129,133], and depletion of intracellular ATP [62,65,71,135]. Membrane integrity can also be assessed by flow cytometry after staining with propidium iodide, a fluorescent dye, alone or in combination with SYTO9. While SYTO9 binds to both dead and living cells, propidium iodide in not able to pass through intact membrane, thus allowing for the detection of injured or damaged cell membranes [65,66,138,139,140]. Terpenes present in EOs are also known to work as efflux pump inhibitors [141]. This ability is usually tested in connection to possible increase of potential of antibiotics due to efflux system being one of mechanisms of antibiotic resistance. The standard method is based on staining with ethidium bromide and fluorescence measurement of the level of its accumulation inside the cells [57,142,143]. Although the impaired function of efflux pumps may be also connected with ATP depletion, down-regulated gene expression of efflux pump components has been observed in multiple studies [57,141,144].

Decreased ATPase and NADH oxidase activity, corroborated by down-regulation of genes participating in oxidative phosphorylation process, has been described, proving that EOs may hamper with energy metabolism of the bacterial cell [135]. Increased oxidative stress by accumulation of intracellular Reactive Oxygen Species (ROS) [62,65,133] and increased extracellular malondialdehyde concentration [62,65] has been reported after application of EO.

Recently, individual components of EO have been studied in silico using PASS (Prediction of activity spectra for substances). The database compares molecules structures and based on the similarity in binding sites predicts the potential biological (in this case antibacterial) activity [138]. Similarly, molecular docking simulations allow for the model binding of the EO component on an active site of a protein of importance [62,98,119,144].

## 9. Methodology

For Figure 1, a search was conducted in the Web of Science database on 7 November 2024. The research topic (Article title, Abstract, and Keywords) was searched using the terms “essential oil” and “*Salmonella*”, respectively. The results for individual years were obtained using the “Refine by Publication Years” option.

For Figure 2, the search was conducted in the Scopus database on 7 November 2024, as the search in the Web of Science tended to include many irrelevant results. The Article title, Abstract, and Keywords were searched using the following strings: “essential oil” AND mic AND (macrodilution OR macro-dilution OR “tube dilution”); “essential oil” AND mic AND (microdilution OR micro-dilution); “essential oil” AND MIC; “essential oil” AND inhibition AND zone; “essential oil” AND MIC AND ?g/mL; “essential oil” AND MIC AND ?L/mL; “essential oil” AND MIC AND “*v*/*v*”. Verification of the search relevance was performed by sorting the results by relevance and checking the five least relevant results. The results on volumetric percentage may be strongly biased, as it is not possible to search for the “%” sign, and not every publication included the “*v*/*v*” specification in the abstract. It should also be noted that some publications may not include specific values (and therefore units) or specific methods in the abstract.

## 10. Conclusions and Prospect

Although only a small part of the relevant literature could be researched in detail, it is clear that there is a great variability in assays testing antimicrobial activity of EOs. Although some variables can be reduced simply by keeping to CLSI standards for testing of antibiotics, there are still some variables to be optimized and standardized given the unique properties of EOs, notably their volatility and hydrophobic nature. Based on the literature research, following suggestions when designing an experiment can be recommended:

1. Chemical composition of the tested EO/EOs should be always made available, since there is a great variability among EOs of the same botanical origin. Note that many journals have already made that mandatory.
2. It is highly recommended to use multiple assays, especially when agar diffusion method is used—this method is useful for preliminary studies but should not stand alone as the only method of investigation.
3. The assays need to be standardized as much as possible, at least in the most important parameters. CLSI guidelines can be recommended for some parameters, such as media, media preparation, inoculum size, and quality control. For the disk diffusion method, a disk size of 6 mm soaked with 10 μL of EO is recommended. For broth dilution, MHB with 0.15% of agar for better dispersion of EO seems to be the most fitting medium, as there is no doubt about its non-toxicity. Use of solvents such as ethanol or DMSO should be further considered and evaluated. If used, the maximum concentration should be stated and tested for toxicity especially when fastidious bacteria are to be tested.
4. MIC should be reported in standardized units such as mg or μg/mL.
5. All tests should be performed in triplicate.

Especially, the miniaturized dilution assays, alone or in combination with vapor phase evaluation, should be further explored, as they seem to have a great potential.

## Figures and Tables

**Table 1:** Solubility of major EOs components in water at 25 °C [80].

Compound	CAS Number	Solubility [mmol/L]	Solubility [mg/L]
α-pinene	80-56-8	0.035–0.039	4.80–5.27
Carvacrol	499-75-2	6.65–8.32	999–1250
Carvone	6485-40-1	8.65–11.0	1300–1652
Citral	5392-40-5	1.58–3.80	241–578
Eucalyptol (1,8-cineole)	470-82-6	20.1–22.0	3100–3388
Eugenol	97-53-0	15.0	2463
γ-Terpinene	99-85-4	0.064	8.68
Geraniol	106-24-1	5.00	771
Limonene	138-86-3	0.064–0.220	8.71–30.0
Linalool	78-70-6	9.71–12.0	1498–1851
Menthol	89-78-1	2.92	456
*p*-Cymene	99-87-6	0.174–2.98	23.3–400
Thymol	89-83-8	5.70–6.65	856–999
Trans-cinnamaldehyde	14371-10-9	14.0	1850

**Table 2:** Recommended attributes for methods used for determination of antimicrobial potential of EOs.

Method	Factors	Recommendation
Broth dilution	Solvent	If necessary, DMSO at final concentration in the assay ≤ 1%. Check for potential inhibitory effect of DMSO on fastidious bacteria.
	Medium	Mueller–Hinton broth
	Stabilizer	0.15% of agar
	Inoculum	approx. 5 × 10^5^ cells/mL in the final assay
	Endpoint	OD measurement or visually; there is no evidence so far that the use of dyes such as resazurin or tetrazolium salts actually results in different MIC values, although they may be helpful in detecting the endpoint.
	MIC units	μg or mg per mL
Agar dilution	Solvent	If necessary, DMSO at final concentration in the assay ≤ 1%. Check for potential inhibitory effect of DMSO on fastidious bacteria.
	Medium	Mueller–Hinton agar
	Plate preparation	3–4 mm in depth. Avoid excessive and prolonged heating of the agar with EO solution, as some EO components are heat sensitive.
	Inoculum	approx. 10^4^ cells per spot
	MIC units	μg or mg per mL
Agar disk diffusion	EO application	10 μL of pure EO
	Disk size	6 mm
	Medium	Mueller–Hinton agar
	Plate preparation	approx. 4 mm in depth
	Inoculum	approx. 10^8^ cells/mL

## References

[1] Franz C., Novak J., Baser K.H.C., Buchbauer G. (2020). Sources of Essential Oils. Handbook of Essential Oils: Science, Technology, and Applications.

[2] Ribeiro-Santos R., Andrade M., Sanches-Silva A., de Melo N.R. (2018). Essential Oils for Food Application: Natural Substances with Established Biological Activities. Food Bioprocess Technol..

[3] Hyldgaard M., Mygind T., Meyer R.L. (2012). Essential Oils in Food Preservation: Mode of Action, Synergies, and Interactions with Food Matrix Components. Front. Microbiol..

[4] de Sousa D.P., Damasceno R.O.S., Amorati R., Elshabrawy H.A., de Castro R.D., Bezerra D.P., Nunes V.R.V., Gomes R.C., Lima T.C. (2023). Essential Oils: Chemistry and Pharmacological Activities. Biomolecules.

[5] Amiri A., Mottaghipisheh J., Jamshidi-Kia F., Saeidi K., Vitalini S., Iriti M. (2020). Antimicrobial Potencies of Major Functional Foods’ Essential Oils in Liquid and Vapor Phases: A Short Review. Appl. Sci..

[6] Reyes-Jurado F., Franco-Vega A., Ramirez-Corona N., Palou E., López-Malo A. (2015). Essential Oils: Antimicrobial Activities, Extraction Methods, and Their Modeling. Food Eng. Rev..

[7] Masyita A., Mustika Sari R., Dwi Astuti A., Yasir B., Rahma Rumata N., Emran T.B., Nainu F., Simal-Gandara J. (2022). Terpenes and Terpenoids as Main Bioactive Compounds of Essential Oils, Their Roles in Human Health and Potential Application as Natural Food Preservatives. Food Chem. X.

[8] Sadgrove N.J., Padilla-González G.F., Phumthum M. (2022). Fundamental Chemistry of Essential Oils and Volatile Organic Compounds, Methods of Analysis and Authentication. Plants.

[9] Burt S. (2004). Essential Oils: Their Antibacterial Properties and Potential Applications in Foods—A Review. Int. J. Food Microbiol..

[10] Ramsey J.T., Shropshire B.C., Nagy T.R., Chambers K.D., Li Y., Korach K.S. (2020). Essential Oils and Health. Yale J. Biol. Med..

[11] Baser K.H.C., Franz C., Baser K.H.C., Buchbauer G. (2020). Essential Oils Used in Veterinary Medicine. Handbook of Essential Oils: Science, Technology, and Applications.

[12] Guzmán E., Lucia A. (2021). Essential Oils and Their Individual Components in Cosmetic Products. Cosmetics.

[13] Nahas R.I., Baines D., Seal R. (2012). Natural antioxidants as food and beverage ingredients. Natural Food Additives, Ingredients and Flavourings.

[14] Delves-Broughton J., Baines D., Seal R. (2012). Natural antimicrobials as additives and ingredients for the preservation of foods and beverages. Natural Food Additives, Ingredients and Flavourings.

[15] Rowe D.J., Baines D., Seal R. (2012). Natural aroma chemicals for use in foods and beverages. Natural Food Additives, Ingredients and Flavourings.

[16] Catani L., Grassi E., di Montanara A.C., Guidi L., Sandulli R., Manachini B., Semprucci F. (2022). Essential Oils and Their Applications in Agriculture and Agricultural Products: A Literature Analysis through VOSviewer. Biocatal. Agric. Biotechnol..

[17] Calo J.R., Crandall P.G., O’Bryan C.A., Ricke S.C. (2015). Essential Oils as Antimicrobials in Food Systems—A Review. Food Control.

[18] WHO (2015). Global Antimicrobial Resistance Surveillance System: Manual for Early Implementation.

[19] Othman M., Loh H.S., Wiart C., Khoo T.J., Lim K.H., Ting K.N. (2011). Optimal Methods for Evaluating Antimicrobial Activities from Plant Extracts. J. Microbiol. Methods.

[20] Balouiri M., Sadiki M., Ibnsouda S.K. (2016). Methods for In Vitro Evaluating Antimicrobial Activity: A Review. J. Pharm. Anal..

[21] Van de Vel E., Sampers I., Raes K. (2019). A Review on Influencing Factors on the Minimum Inhibitory Concentration of Essential Oils. Crit. Rev. Food Sci. Nutr..

[22] Rao J., Chen B., McClements D.J. (2019). Improving the Efficacy of Essential Oils as Antimicrobials in Foods: Mechanisms of Action. Annu. Rev. Food Sci. Technol..

[23] Mihajilov-Krstev T., Radnovic D., Kitic D., Stojanovic-Radic Z., Zlatkovic B. (2009). Antimicrobial Activity of *Satureja hortensis* L. Essential Oil Against Pathogenic Microbial Strains. Biotechnol. Biotechnol. Equip..

[24] Hussain A.I., Anwar F., Nigam P.S., Sarker S.D., Moore J.E., Rao J.R., Mazumdar A. (2011). Antibacterial Activity of Some Lamiaceae Essential Oils Using Resazurin as an Indicator of Cell Growth. LWT—Food Sci. Technol..

[25] Jadhav S., Shah R., Bhave M., Palombo E.A. (2013). Inhibitory Activity of Yarrow Essential Oil on *Listeria* Planktonic Cells and Biofilms. Food Control.

[26] Miladinović D.L., Ilić B.S., Mihajilov-Krstev T.M., Nikolić D.M., Cvetković O.G., Marković M.S., Miladinović L.C. (2013). Antibacterial Activity of the Essential Oil of *Heracleum sibiricum*. Nat. Prod. Commun..

[27] Bazargani M.M., Rohloff J. (2016). Antibiofilm Activity of Essential Oils and Plant Extracts Against *Staphylococcus aureus* and *Escherichia coli* Biofilms. Food Control.

[28] Puškárová A., Bučková M., Kraková L., Pangallo D., Kozics K. (2017). The Antibacterial and Antifungal Activity of Six Essential Oils and Their Cyto/Genotoxicity to Human HEL 12469 Cells. Sci. Rep..

[29] Thielmann J., Muranyi P., Kazman P. (2019). Screening Essential Oils for Their Antimicrobial Activities Against the Foodborne Pathogenic Bacteria *Escherichia coli* and *Staphylococcus aureus*. Heliyon.

[30] El Hachlafi N., Mrabti H.N., Al-Mijalli S.H., Jeddi M., Abdallah E.M., Benkhaira N., Hadni H., Assaggaf H., Qasem A., Goh K.W. (2023). Antioxidant, Volatile Compounds; Antimicrobial, Anti-Inflammatory, and Dermatoprotective Properties of *Cedrus atlantica* (Endl.) Manetti Ex Carriere Essential Oil: In Vitro and In Silico Investigations. Molecules.

[31] Mrabti H.N., El Hachlafi N., Al-Mijalli S.H., Jeddi M., Elbouzidi A., Abdallah E.M., Flouchi R., Assaggaf H., Qasem A., Zengin G. (2023). Phytochemical Profile, Assessment of Antimicrobial and Antioxidant Properties of Essential Oils of *Artemisia herba-alba Asso.*, and *Artemisia dracunculus* L.: Experimental and Computational Approaches. J. Mol. Struct..

[32] Pellegrini M., Ricci A., Serio A., Chaves-López C., Mazzarrino G., D’Amato S., Lo Sterzo C., Paparella A. (2018). Characterization of Essential Oils Obtained from Abruzzo Autochthonous Plants: Antioxidant and Antimicrobial Activities Assessment for Food Application. Foods.

[33] Houdkova M., Chaure A., Doskocil I., Havlik J., Kokoska L. (2021). New Broth Macrodilution Volatilization Method for Antibacterial Susceptibility Testing of Volatile Agents and Evaluation of Their Toxicity Using Modified MTT Assay In Vitro. Molecules.

[34] Hulankova R. (2022). Higher Resistance of *Yersinia enterocolitica* in Comparison to *Yersinia pseudotuberculosis* to Antibiotics and Cinnamon, Oregano and Thyme Essential Oils. Pathogens.

[35] Ostrosky E.A., Mizumoto M.K., Lima M.E.L., Kaneko T.M., Nishikawa S.O., Freitas B.R. (2008). Methods for Evaluation of the Antimicrobial Activity and Determination of Minimum Inhibitory Concentration (MIC) of Plant Extracts. Rev. Bras. Farmacogn..

[36] Wiegand I., Hilpert K., Hancock R.E.W. (2008). Agar and Broth Dilution Methods to Determine the Minimal Inhibitory Concentration (MIC) of Antimicrobial Substances. Nat. Protoc..

[37] (2018). Methods for Dilution Antimicrobial Susceptibility Tests for Bacteria That Grow Aerobically, 11th ed.

[38] Zhao A., Zhang Y., Li F., Chen L., Huang X. (2023). Analysis of the Antibacterial Properties of Compound Essential Oil and the Main Antibacterial Components of Unilateral Essential Oils. Molecules.

[39] Cervenka L., Peskova I., Pejchalova M., Vytrasova J. (2008). Inhibition of *Arcobacter butzleri*, *Arcobacter cryaerophilus*, and *Arcobacter skirrowii* by Plant Oil Aromatics. J. Food Prot..

[40] Donaldson J.R., Warner S.L., Cates R.G., Young D.G. (2005). Assessment of Antimicrobial Activity of Fourteen Essential Oils When Using Dilution and Diffusion Methods. Pharm. Biol..

[41] Sakkas H., Economou V., Gousia P., Bozidis P., Sakkas V.A., Petsios S., Mpekoulis G., Ilia A., Papadopoulou C. (2018). Antibacterial Efficacy of Commercially Available Essential Oils Tested Against Drug-Resistant Gram-Positive Pathogens. Appl. Sci..

[42] de Azeredo G.A., Stamford T.L.M., Nunes P.C., Neto N.J.G., de Oliveira M.E.C., de Souza E.L. (2011). Combined Application of Essential Oils from *Origanum vulgare* L. and *Rosmarinus officinalis* L. to Inhibit Bacteria and Autochthonous Microflora Associated with Minimally Processed Vegetables. Food Res. Int..

[43] Porter J.A., Monu E.A. (2019). Evaluating the Antimicrobial Efficacy of White Mustard Essential Oil Alone and in Combination with Thymol and Carvacrol Against *Salmonella*. J. Food Prot..

[44] Kavanagh A., Ramu S., Gong Y., Cooper M.A., Blaskovich M.A.T. (2019). Effects of Microplate Type and Broth Additives on Microdilution MIC Susceptibility Assays. Antimicrob. Agents Chemother..

[45] (2019). Susceptibility Testing of Infectious Agents and Evaluation of Performance of Antimicrobial Susceptibility Test Devices Part 1: Broth Micro-Dilution Reference Method for Testing the In Vitro Activity of Antimicrobial Agents against Rapidly Growing Aerobic Bacteria Involved in Infectious Diseases.

[46] Vanegas D., Abril-Novillo A., Khachatryan A., Jerves-Andrade L., Peñaherrera E., Cuzco N., Wilches I., Calle J., León-Tamariz F. (2021). Validation of a Method of Broth Microdilution for the Determination of Antibacterial Activity of Essential Oils. BMC Res. Notes.

[47] Seow Y.X., Yeo C.R., Chung H.L., Yuk H.G. (2014). Plant Essential Oils as Active Antimicrobial Agents. Crit. Rev. Food Sci. Nutr..

[48] Klančnik A., Piskernik S., Jeršek B., Smole Možina S. (2010). Evaluation of Diffusion and Dilution Methods to Determine the Antibacterial Activity of Plant Extracts. J. Microbiol. Methods.

[49] Kavanaugh N.L., Ribbeck K. (2012). Selected Antimicrobial Essential Oils Eradicate *Pseudomonas* spp. and *Staphylococcus aureus* Biofilms. Appl. Environ. Microbiol..

[50] Boukhira S., Balouiri M., Bousta F., Moularat S., Taleb M.S., Bousta D. (2016). Antimicrobial Activities of Essential Oil of Five Plant Species from Morocco Against Some Microbial Strains. Int. J. Pharmacogn. Pharm. Res..

[51] Park J.W., Wendt M., Heo G.J. (2016). Antimicrobial Activity of Essential Oil of *Eucalyptus globulus* Against Fish Pathogenic Bacteria. Lab Anim. Res..

[52] Alexopoulos A., Plessas S., Kimbaris A., Varvatou M., Mantzourani I., Fournomiti M. (2017). Mode of Antimicrobial Action of *Origanum vulgare* Essential Oil Against Clinical Pathogens. Curr. Res. Nutr. Food Sci..

[53] Sadiki F.Z., El Idrissi M., Sbiti M., Lemrhari A., Trifan A., Cioanca O., Postu P.A., Hritcu L. (2018). Chemical Composition and Antibacterial Activity of Essential Oil of *Tetraclinis articulata* (Vahl) Masters Branches of Eastern Morocco. Chem. Biol. Technol. Agric..

[54] Gonçalves G.M.S., Barros P.P., Silva G.H., Fedes G.R. (2019). The Essential Oil of *Curcuma longa* Rhizomes as an Antimicrobial and Its Composition by CG-MS. Rev. Ciênc. Med..

[55] Rathore S., Mukhia S., Kapoor S., Bhatt V., Kumar R., Kumar R. (2022). Seasonal Variability in Essential Oil Composition and Biological Activity of *Rosmarinus officinalis* L. Accessions in the Western Himalaya. Sci. Rep..

[56] Van N.T.B., Vi O.T., Yen N.T.P., Nhung N.T., Cuong N.V., Kiet B.T., Hoang N.V., Hien V.B., Thwaites G., Campbell J. (2022). Minimum Inhibitory Concentrations of Commercial Essential Oils Against Common Chicken Pathogenic Bacteria and Their Relationship with Antibiotic Resistance. J. Appl. Microbiol..

[57] Abdelatti M.A.I., Abd El-Aziz N.K., El-Naenaeey E.Y.M., Ammar A.M., Alharbi N.K., Alharthi A., Zakai S.A., Abdelkhalek A. (2023). Antibacterial and Anti-Efflux Activities of Cinnamon Essential Oil Against Pan and Extensive Drug-Resistant *Pseudomonas aeruginosa* Isolated from Human and Animal Sources. Antibiotics.

[58] Chen S., Li Z., Gu Z., Ban X., Hong Y., Cheng L., Li C. (2023). A New Micro-Agar Dilution Method to Determine the Minimum Inhibitory Concentration of Essential Oils Against Microorganisms. J. Microbiol. Methods.

[59] Cui Z.H., He H.L., Wu S.B., Dong C.L., Lu S.Y., Shan T.J., Fang L.X., Liao X.P., Liu Y.H., Sun J. (2021). Rapid Screening of Essential Oils as Substances Which Enhance Antibiotic Activity Using a Modified Well Diffusion Method. Antibiotics.

[60] EUCAST (2020). Media Preparation for EUCAST Disk Diffusion Testing and for Determination of MIC Values by the Broth Microdilution Method.

[61] Rota C., Carramiñana J.J., Burillo J., Herrera A. (2004). In Vitro Antimicrobial Activity of Essential Oils from Aromatic Plants Against Selected Foodborne Pathogens. J. Food Prot..

[62] Guo P., Li Z., Cai T., Guo D., Yang B., Zhang C., Shan Z., Wang X., Peng X., Liu G. (2024). Inhibitory effect and mechanism of oregano essential oil on *Listeria monocytogenes* cells, toxins and biofilms. Microb. Pathog..

[63] Raeisi M., Tajik H., Yarahmadi A., Sanginabadi S. (2015). Antimicrobial Effect of Cinnamon Essential Oil Against *Escherichia coli* and *Staphylococcus aureus*. Health Scope.

[64] Knezevic P., Aleksic Sabo V., Simin N., Lesjak M., Mimica-Dukic N. (2018). A Colorimetric Broth Microdilution Method for Assessment of *Helicobacter pylori* Sensitivity to Antimicrobial Agents. J. Pharm. Biomed. Anal..

[65] Zhan X., Tan Y., Lv Y., Fang J., Zhou Y., Gao X., Zhu H., Shi C. (2022). The Antimicrobial and Antibiofilm Activity of Oregano Essential Oil against *Enterococcus faecalis* and Its Application in Chicken Breast. Foods.

[66] Gędas A., Draszanowska A., den Bakker H., Diez-Gonzalez F., Simões M., Olszewska M.A. (2023). Prevention of surface colonization and anti-biofilm effect of selected phytochemicals against *Listeria innocua* strain. Colloids Surf. B Biointerfaces.

[67] Hsaine S., Charof R., Ounine K. (2017). Evaluation of Antibacterial Activity of Essential Oil of *Cinnamomum zeylanicum*, *Eugenia caryophyllata*, and *Rosmarinus officinalis* Against *Streptococcus oralis*. Asian J. Pharm. Clin. Res..

[68] Bouyahya A., Abrini J., Dakka N., Bakri Y. (2019). Essential Oils of *Origanum compactum* Increase Membrane Permeability, Disturb Cell Membrane Integrity, and Suppress Quorum-Sensing Phenotype in Bacteria. J. Pharm. Anal..

[69] Cazella L.N., Glamoclija J., Soković M., Gonçalves J.E., Linde G.A., Colauto N.B., Gazim Z.C. (2019). Antimicrobial Activity of Essential Oil of *Baccharis dracunculifolia* DC (Asteraceae) Aerial Parts at Flowering Period. Front. Plant Sci..

[70] Sateriale D., Forgione G., De Cristofaro G.A., Facchiano S., Boscaino F., Pagliuca C., Colicchio R., Salvatore P., Paolucci M., Pagliarulo C. (2022). Towards Green Strategies of Food Security: Antibacterial Synergy of Essential Oils from *Thymus vulgaris* and *Syzygium aromaticum* to Inhibit *Escherichia coli* and *Staphylococcus aureus* Pathogenic Food Isolates. Microorganisms.

[71] Cai T., Li Z., Guo P., Guo J., Wang R., Guo D., Yu J., Lü X., Xia X., Shi C. (2023). Antimicrobial and Antibiofilm Efficacy and Mechanism of Oregano Essential Oil Against *Shigella flexneri*. Foodborne Pathog. Dis..

[72] Tapia-Rodriguez M.R., Cantu-Soto E.U., Vazquez-Armenta F.J., Bernal-Mercado A.T., Ayala-Zavala J.F. (2023). Inhibition of *Acinetobacter baumannii* Biofilm Formation by Terpenes from Oregano (*Lippia graveolens*) Essential Oil. Antibiotics.

[73] Zhu W.X., Li J.H., Tan J.Q., Gong M.M., Wang A.L., Liang C.C., Wang H.S., Xia X.D. (2025). Inhibition of *Shewanella putrefaciens* biofilm by laurel essential oil and its potential mechanisms. Food Control.

[74] Iacovelli F., Romeo A., Lattanzio P., Ammendola S., Battistoni A., La Frazia S., Vindigni G., Unida V., Biocca S., Gaziano R. (2023). Deciphering the Broad Antimicrobial Activity of *Melaleuca alternifolia* Tea Tree Oil by Combining Experimental and Computational Investigations. Int. J. Mol. Sci..

[75] de Barros J.C., da Conceição M.L., Neto N.J., da Costa A.C., de Souza E.L. (2012). Combination of *Origanum vulgare* L. Essential Oil and Lactic Acid to Inhibit *Staphylococcus aureus* in Meat Broth and Meat Model. Braz. J. Microbiol..

[76] Nagalakshmi S., Saranraj P., Sivasakthivelan P. (2019). Determination of Minimum Inhibitory Concentration (MIC) and Percentage Bacterial Growth Inhibition of Essential Oils Against Gram Positive Bacterial Pathogens. J. Drug Deliv. Ther..

[77] Liu J.X., Huang D.F., Hao D.L., Hu Q.P. (2014). Chemical Composition, Antibacterial Activity of the Essential Oil from Roots of *Radix aucklandiae* Against Selected Food-Borne Pathogens. Adv. Biosci. Biotechnol..

[78] Araby E., El-Tablawy S.Y. (2016). Inhibitory Effects of *Rosemary* (*Rosmarinus officinalis* L.) Essential Oil on Pathogenicity of Irradiated and Non-Irradiated *Pseudomonas aeruginosa*. J. Photochem. Photobiol. B.

[79] Hulankova R. (2022). The Influence of Liquid Medium Choice in Determination of Minimum Inhibitory Concentration of Essential Oils Against Pathogenic Bacteria. Antibiotics.

[80] Yalkowsky S.H., He Y., Jain P. (2010). Handbook of Aqueous Solubility Data.

[81] Ilić B.S., Kocić B.D., Cirić V.M., Ćvetković O.G., Miladinović D.L. (2014). An In Vitro Synergistic Interaction of Combinations of *Thymus glabrescens* Essential Oil and Its Main Constituents with Chloramphenicol. Sci. World J..

[82] dal Pozzo M., Silva Loreto E., Flores Santurio D., Hartz Alves S., Rossatto L., Castagna de Vargas A., Viegas J., Matiuzzi da Costa M. (2012). Antibacterial Activity of Essential Oil of Cinnamon and Trans-Cinnamaldehyde Against *Staphylococcus* spp. Isolated from Clinical Mastitis of Cattle and Goats. Acta Sci. Vet..

[83] Wadhwani T., Desai K., Patel D., Lawani D., Bahaley P., Joshi P., Vijay K. (2018). Effect of Various Solvents on Bacterial Growth in Context of Determining MIC of Various Antimicrobials. Internet J. Microbiol..

[84] Tadtong S., Suppawat S., Tintawee A., Saramas P., Jareonvong S., Hongratanaworakit T. (2012). Antimicrobial Activity of Blended Essential Oil Preparation. Nat. Prod. Commun..

[85] Alitonou G.A., Sessou P., Tchobo P.F., Noudogbessi J.P., Avlessi F., Yehouenou B., Menut C., Villeneuve P., Sohounhloue D.C.K. (2012). Chemical Composition and Biological Activities of Essential Oils of *Chenopodium ambrosioides* L. Collected in Two Areas of Benin. Int. J. Biosci..

[86] Nielsen C.K., Kjems J., Mygind T., Snabe T., Meyer R.L. (2016). Effects of Tween 80 on Growth and Biofilm Formation in Laboratory Media. Front. Microbiol..

[87] Tovidé S.N., Adeoti K., Yèhouénou B., Dahouénon-Ahoussi E., Baba-Moussa F., Toukourou F. (2016). Antimicrobial and Physico-Chemical Effects of Essential Oil on Fermented Milk During Preservation. J. Appl. Biosci..

[88] da Silva B.D., do Rosário D.K.A., Neto L.T., Lelis C.A., Conte-Junior C.A. (2023). Antioxidant, Antibacterial and Antibiofilm Activity of Nanoemulsion-Based Natural Compound Delivery Systems Compared with Non-Nanoemulsified Versions. Foods.

[89] Anwer M.K., Jamil S., Ibnouf E.O., Shakeel F. (2014). Enhanced Antibacterial Effects of Clove Essential Oil by Nanoemulsion. J. Oleo Sci..

[90] Pimple V.V., Kulkarni A.S., Patil S.P., Dhoble S.J. (2019). Plant Essential Oils Based Nanoemulsion Formulations and Its Antibacterial Effect on Some Pathogens. Int. J. Innov. Technol. Explor. Eng..

[91] Remmal A., Bouchikhi T., Tantaoui-Elaraki A., Ettayebi M. (1993). Inhibition of Antibacterial Activity of Essential Oils by Tween 80 and Ethanol in Liquid Medium. J. Pharm. Belg..

[92] Mann C.M., Markham J.L. (1998). A New Method for Determining the Minimum Inhibitory Concentration of Essential Oils. J. Appl. Microbiol..

[93] Weseler A., Geiss H.K., Saller R., Reichling J. (2005). A Novel Colorimetric Broth Microdilution Method to Determine the Minimum Inhibitory Concentration (MIC) of Antibiotics and Essential Oils Against *Helicobacter pylori*. Pharmazie.

[94] Sarker S.D., Nahar L., Kumarasamy Y. (2007). Microtitre Plate-Based Antibacterial Assay Incorporating Resazurin as an Indicator of Cell Growth, and Its Application in the In Vitro Antibacterial Screening of Phytochemicals. Methods.

[95] Braissant O., Astasov-Frauenhoffer M., Waltimo T., Bonkat G. (2020). A Review of Methods to Determine Viability, Vitality, and Metabolic Rates in Microbiology. Front. Microbiol..

[96] Eloff J.N. (2019). Avoiding Pitfalls in Determining Antimicrobial Activity of Plant Extracts and Publishing the Results. BMC Complement. Altern. Med..

[97] Zarai Z., Ben Chobba I., Ben Mansour R., Békir A., Gharsallah N., Kadri A. (2012). Essential Oil of the Leaves of *Ricinus communis* L.: In Vitro Cytotoxicity and Antimicrobial Properties. Lipids Health Dis..

[98] Nouioura G., El Fadili M., El Hachlafi N., Abuelizz H.A., Elidrissi A.E., Ferioun M., Soulo N., Er-Rahmani S., Lyoussi B., Derwich E. (2024). *Petroselinum crispum* L., essential oil as promising source of bioactive compounds, antioxidant, antimicrobial activities: In vitro and in silico predictions. Heliyon.

[99] Yèhouenou B., Wotto D.V., Sessou P., Noudogbessi J.P., Sohounhloue D.C.K. (2010). Chemical Study and Antimicrobial Activities of Volatile Extracts from Fresh Leaves of *Crassocephalum rubens* (Juss and Jack.) S. Moore Against Food Borne Pathogens. Sci. Study Res.Chem. Chem. Eng. Biotechnol. Food Ind..

[100] Szweda P., Zalewska M., Pilch J., Kot B., Milewski S. (2018). Essential Oils as Potential Anti-Staphylococcal Agents. Acta Vet..

[101] Kačániová M., Vukic M., Vukovic N.L., Čmiková N., Verešová A., Schwarzová M., Babošová M., Porhajašová J.I., Kluz M., Waszkiewicz-Robak B. (2024). An In-Depth Study on the Chemical Composition and Biological Effects of *Pelargonium graveolens* Essential Oil. Foods.

[102] d’Acampora Zellner B., Dugo P., Dugo G., Mondello L., Baser K.H.C., Buchbauer G. (2020). Analysis of Essential Oils. Handbook of Essential Oils: Science, Technology, and Applications.

[103] Ríos J.L., Preedy V.R. (2016). Essential Oils: What They Are and How the Terms Are Used and Defined. Essential Oils in Food Preservation, Flavor and Safety.

[104] Golus J., Sawicki R., Widelski J., Ginalska G. (2016). The Agar Microdilution Method—A New Method for Antimicrobial Susceptibility Testing for Essential Oils and Plant Extracts. J. Appl. Microbiol..

[105] Fei P., Xu Y., Zhao S., Gong S., Guo L. (2019). Olive Oil Polyphenol Extract Inhibits Vegetative Cells of *Bacillus cereus* Isolated from Raw Milk. J. Dairy Sci..

[106] Liu M., Pan Y., Feng M., Guo W., Fan X., Feng L., Huang J., Cao Y. (2022). Garlic Essential Oil in Water Nanoemulsion Prepared by High-Power Ultrasound: Properties, Stability, and Its Antibacterial Mechanism Against MRSA Isolated from Pork. Ultrason. Sonochem..

[107] López E.I.C., Balcázar M.F.H., Mendoza J.M.R., Ortiz A.D.R., Melo M.T.O., Parrales R.S., Delgado T.H. (2017). Antimicrobial Activity of Essential Oil of *Zingiber officinale* Roscoe (Zingiberaceae). Am. J. Plant Sci..

[108] Ramezani M., Behravan J., Yazdinezhad A. (2005). Chemical Composition and Antimicrobial Activity of the Volatile Oil of *Artemisia khorassanica* from Iran. Pharm. Biol..

[109] Prabuseenivasan S., Jayakumar M., Ignacimuthu S. (2006). In Vitro Antibacterial Activity of Some Plant Essential Oils. BMC Complement. Altern. Med..

[110] Fraternale D., Genovese S., Ricci D. (2013). Essential Oil Composition and Antimicrobial Activity of Aerial Parts and Ripe Fruits of *Echinophora spinosa* (Apiaceae) from Italy. Nat. Prod. Commun..

[111] Shi C., Song K., Zhang X., Sun Y., Sui Y., Chen Y., Jia Z., Sun H., Sun Z., Xia X. (2016). Antimicrobial Activity and Possible Mechanism of Action of Citral Against *Cronobacter sakazakii*. PLoS ONE.

[112] Sun Y., Guo D., Hua Z., Sun H., Zheng Z., Xia X., Shi C. (2019). Attenuation of Multiple *Vibrio parahaemolyticus* Virulence Factors by Citral. Front. Microbiol..

[113] Nikolić I., Chua E.G., Tay A.C.Y., Kostrešević A., Pavlović B., Jončić Savić K. (2023). Savory, Oregano, and Thyme Essential Oil Mixture (HerbELICO^®^) Counteracts *Helicobacter pylori*. Molecules.

[114] Selim S. (2011). Antimicrobial Activity of Essential Oils Against Vancomycin-Resistant Enterococci (VRE) and *Escherichia coli* O157 in Feta Soft Cheese and Minced Beef Meat. Braz. J. Microbiol..

[115] Valgas C., de Souza S.M., Smânia E.F.A., Smânia A. (2007). Screening Methods to Determine Antibacterial Activity of Natural Products. Braz. J. Microbiol..

[116] Ponce A.G., Fritz R., Del Valle C., Roura S.I. (2003). Antimicrobial Activity of Essential Oils on the Native Microflora of Organic Swiss Chard. Lebensm. Wiss. Technol..

[117] Rota M.C., Herrera A., Martínez R.M., Sotomayor J.A., Jordán M.J. (2008). Antimicrobial Activity and Chemical Composition of *Thymus vulgaris*, *Thymus zygis*, and *Thymus hyemalis* Essential Oils. Food Control.

[118] (2024). Performance Standards for Antimicrobial Disk Susceptibility Tests, 14th ed.

[119] Yuan Y., Sun J., Song Y., Raka R.N., Xiang J., Wu H., Xiao J., Jin J., Hui X. (2023). Antibacterial activity of oregano essential oils against *Streptococcus mutans* in vitro and analysis of active components. BMC Complement. Med. Ther..

[120] Ignatiuk K., Dzikon E., Hagdej B., Slotwinska W., Malm M., Ossowski M., Kasela M. (2023). Comparison of Disc-Diffusion and Disc-Volatilization Assays for Determining the Antimicrobial Activity of *Thymus vulgaris* L. Essential Oil. Curr. Issues Pharm. Med. Sci..

[121] Houdkova M., Kokoska L. (2020). Volatile Antimicrobial Agents and In Vitro Methods for Evaluating Their Activity in the Vapour Phase: A Review. Planta Med..

[122] Laird K., Phillips C. (2012). Vapour Phase: A Potential Future Use for Essential Oils as Antimicrobials?. Lett. Appl. Microbiol..

[123] López P., Sánchez C., Batlle R., Nerín C. (2005). Solid- and Vapour-Phase Antimicrobial Activities of Six Essential Oils: Susceptibility of Selected Foodborne Bacterial and Fungal Strains. J. Agric. Food Chem..

[124] Ács K., Balázs V.L., Kocsis B., Bencsik T., Böszörményi A., Horváth G. (2018). Antibacterial Activity Evaluation of Selected Essential Oils in Liquid and Vapor Phase on Respiratory Tract Pathogens. BMC Complement. Altern. Med..

[125] Kloucek P., Smid J., Frankova A., Kokoska L., Valterova I., Pavela R. (2012). Fast Screening Method for Assessment of Antimicrobial Activity of Essential Oils in Vapour Phase. Food Res. Int..

[126] Houdkova M., Rondevaldova J., Doskocil I., Kokoska L. (2017). Evaluation of Antibacterial Potential and Toxicity of Plant Volatile Compounds Using New Broth Microdilution Volatilization Method and Modified MTT Assay. Fitoterapia.

[127] Han A., Lee S.Y. (2023). An Overview of Various Methods for In Vitro Biofilm Formation: A Review. Food Sci. Biotechnol..

[128] Cui H.Y., Zhang C.H., Li C.Z., Lin L. (2020). Inhibition mechanism of cardamom essential oil on methicillin-resistant Staphylococcus aureus biofilm. LWT—Food Sci. Technol..

[129] Zhang C., Li C., Abdel-Samie M.A., Cui H., Li C. (2020). Unraveling the Inhibitory Mechanism of Clove Essential Oil Against *Listeria monocytogenes* Biofilm and Applying It to Vegetable Surfaces. LWT—Food Sci. Technol..

[130] Nuță D.C., Limban C., Chiriță C., Chifiriuc M.C., Costea T., Ioniță P., Nicolau I., Zarafu I. (2021). Contribution of Essential Oils to the Fight against Microbial Biofilms—A Review. Processes.

[131] Nourbakhsh F., Nasrollahzadeh M.S., Tajani A.S., Soheili V., Hadizadeh F. (2022). Bacterial biofilms and their resistance mechanisms: A brief look at treatment with natural agents. Folia Microbiol..

[132] Artini M., Papa R., Sapienza F., Božović M., Vrenna G., Tuccio Guarna Assanti V., Sabatino M., Garzoli S., Fiscarelli E.V., Ragno R. (2022). Essential Oils Biofilm Modulation Activity and Machine Learning Analysis on *Pseudomonas aeruginosa* Isolates from Cystic Fibrosis Patients. Microorganisms.

[133] Liu T., Kang J., Liu L. (2021). Thymol as a Critical Component of *Thymus vulgaris* L Essential Oil Combats *Pseudomonas aeruginosa* by Intercalating DNA and Inactivating Biofilm. LWT—Food Sci. Technol..

[134] Azeredo J., Azevedo N.F., Briandet R., Cerca N., Coenye T., Costa A.R., Desvaux M., Di Bonaventura G., Hébraud M., Jaglic Z. (2017). Critical Review on Biofilm Methods. Crit. Rev. Microbiol..

[135] Liu Y., Yan Y., Yang K., Yang X., Dong P., Wu H., Luo X., Zhang Y., Zhu L. (2023). Inhibitory mechanism of Salmonella Derby biofilm formation by sub-inhibitory concentrations of clove and oregano essential oil: A global transcriptomic study. Food Control.

[136] Moghadam M.J., Maktabi S., Zarei M., Mahmoodi P. (2024). Controlling *Staphylococcus aureus* biofilm on food contact surfaces: The efficacy of *Oliveria decumbens* essential oil and its implications on biofilm-related genes. J. Appl. Microbiol..

[137] Reyes-Jurado M., Munguía-Pérez R., Cid-Pérez T.S., Hernández-Carranza P., Ochoa-Velasco C.E., Avila-Sosa R. (2020). Inhibitory Effect of Mexican Oregano (*Lippia berlandieri Schauer*) Essential Oil on *Pseudomonas aeruginosa* and *Salmonella Thyphimurium* Biofilm Formation. Front. Sustain. Food Syst..

[138] Bautista-Silva J.P., Seibert J.B., Amparo T.R., Rodrigues I.V., Teixeira L.F.M., Souza G.H.B., Dos Santos O.D.H. (2020). *Melaleuca leucadendra* Essential Oil Promotes Loss of Cell Membrane and Wall Integrity and Inhibits Bacterial Growth: An In Silico and In Vitro Approach. Curr. Microbiol..

[139] Gómez-García M., Argüello H., Puente H., Mencía-Ares Ó., González S., Miranda R., Rubio P., Carvajal A. (2020). In-depth in vitro Evaluation of the Activity and Mechanisms of Action of Organic Acids and Essential Oils Against Swine Enteropathogenic Bacteria. Front. Vet. Sci..

[140] Wang L., Zhang K., Zhang K., Zhang J., Fu J., Li J., Wang G., Qiu Z., Wang X., Li J. (2020). Antibacterial Activity of *Cinnamomum camphora* Essential Oil on *Escherichia coli* During Planktonic Growth and Biofilm Formation. Front. Microbiol..

[141] Dias K.J.S.O., Miranda G.M., Bessa J.R., Araújo A.C.J., Freitas P.R., Almeida R.S., Paulo C.L.R., Neto J.B.A., Coutinho H.D.M., Ribeiro-Filho J. (2022). Terpenes as bacterial efflux pump inhibitors: A systematic review. Front. Pharmacol..

[142] da Silva L.Y.S., Paulo C.L.R., Moura T.F. (2023). Antibacterial Activity of the Essential Oil of *Piper tuberculatum* Jacq. Fruits against Multidrug-Resistant Strains: Inhibition of Efflux Pumps and β-Lactamase. Plants.

[143] Ghazal T.S.A., Schelz Z., Vidács L., Szemerédi N., Veres K., Spengler G., Hohmann J. (2022). Antimicrobial, Multidrug Resistance Reversal and Biofilm Formation Inhibitory Effect of *Origanum majorana* Extracts, Essential Oil and Monoterpenes. Plants.

[144] Tomaś N., Myszka K., Wolko Ł., Nuc K., Szwengiel A., Grygier A., Majcher M. (2021). Effect of black pepper essential oil on quorum sensing and efflux pump systems in the fish-borne spoiler *Pseudomonas psychrophila* KM02 identified by RNA-seq, RT-qPCR and molecular docking analyses. Food Control.

